# Implementation of a multi-site neonatal simulation improvement program: a cost analysis

**DOI:** 10.1186/s12913-024-11075-z

**Published:** 2024-05-14

**Authors:** Xiao Xu, John Yao, Janine Bohnert, Nicole Yamada, Henry C. Lee

**Affiliations:** 1 Department of Obstetrics and Gynecology, Columbia University Vagelos College of Physicians and Surgeons, New York, NY USA; 2 Department of Obstetrics, Gynecology and Reproductive Sciences, Yale University School of Medicine, New Haven, CT USA; 3 Department of Pediatrics, Stanford University School of Medicine, Stanford, CA USA; 4 Department of Pediatrics, University of California San Diego School of Medicine, 9300 Campus Point Drive, MC 7774, La Jolla, CA 92037 USA

**Keywords:** Neonatal resuscitation program, In situ simulation, Simulation science, Cost analysis

## Abstract

**Background:**

To improve patient outcomes and provider team practice, the California Perinatal Quality Care Collaborative (CPQCC) created the Simulating Success quality improvement program to assist hospitals in implementing a neonatal resuscitation training curriculum. This study aimed to examine the costs associated with the design and implementation of the Simulating Success program.

**Methods:**

From 2017–2020, a total of 14 sites participated in the Simulating Success program and 4 of them systematically collected resource utilization data. Using a micro-costing approach, we examined costs for the design and implementation of the program occurring at CPQCC and the 4 study sites. Data collection forms were used to track personnel time, equipment/supplies, space use, and travel (including transportation, food, and lodging). Cost analysis was conducted from the healthcare sector perspective. Costs incurred by CPQCC were allocated to participant sites and then combined with site-specific costs to estimate the mean cost per site, along with its 95% confidence interval (CI). Cost estimates were inflation-adjusted to 2022 U.S. dollars.

**Results:**

Designing and implementing the Simulating Success program cost $228,148.36 at CPQCC, with personnel cost accounting for the largest share (92.2%), followed by program-related travel (6.1%), equipment/supplies (1.5%), and space use (0.2%). Allocating these costs across participant sites and accounting for site-specific resource utilizations resulted in a mean cost of $39,210.69 per participant site (95% CI: $34,094.52-$44,326.86). In sensitivity analysis varying several study assumptions (e.g., number of participant sites, exclusion of design costs, and useful life span of manikins), the mean cost per site changed from $35,645.22 to $39,935.73. At all four sites, monthly cost of other neonatal resuscitation training was lower during the program implementation period (mean = $1,112.52 per site) than pre-implementation period (mean = $2,504.01 per site). In the 3 months after the Simulating Success program ended, monthly cost of neonatal resuscitation training was also lower than the pre-implementation period at two of the four sites.

**Conclusions:**

Establishing a multi-site neonatal in situ simulation program requires investment of sufficient resources. However, such programs may have financial and non-financial benefits in the long run by offsetting the need for other neonatal resuscitation training and improving practice.

**Supplementary Information:**

The online version contains supplementary material available at 10.1186/s12913-024-11075-z.

## Contributions to the literature

- Despite ample reports on simulation-based training programs in healthcare, data on resource utilization in the implementation of such programs are sparse.
- This study presents an in-depth analysis of the costs involved in the design and delivery of Simulating Success – an evidence-based, multi-hospital neonatal resuscitation training program in the setting of neonatal intensive care units.
- The findings suggest that implementation of the Simulating Success program was associated with substantial costs; however, there may be financial and non-financial benefits in the long run.

## Introduction

Neonatal resuscitation is a critical, albeit low-frequency event that necessitates high levels of individual and teamwork skills. Patient harm and medical errors may occur due to suboptimal communication, lack of adherence to neonatal resuscitation guidelines, and other systems issues [1]. Simulation-based debriefing for healthcare professionals may facilitate teamwork and improve patient outcomes [2, 3]. On-site simulations or in situ simulations that take place in workplace settings allow for realistic and educational scenarios where healthcare teams are encouraged to uncover individual or team weaknesses, system errors, and latent safety threats that can prompt changes in practice. Despite several reports on such simulation programs, there has been little data on the costs and resources involved in their implementation [4–8].

The California Perinatal Quality Care Collaborative (CPQCC) developed the Simulating Success quality improvement program to help California hospitals implement in situ simulation-based neonatal resuscitation training [9, 10]. The Simulating Success program consisted of online didactic training modules to review the foundations of simulation-based training as an educational methodology; face-to-face training on the core principles of developing, conducting, and debriefing simulation-based training; recurrent expert debriefings of in situ simulations at participant sites with monthly online check-ins; and follow-up site visits to provide continued feedback and support. This study aimed to examine the costs associated with the design and implementation of the Simulating Success program. The experience shared here may inform future implementation of other in situ simulation-based quality improvement programs.

## Methods

### Simulating success program

**Fig. 1:**
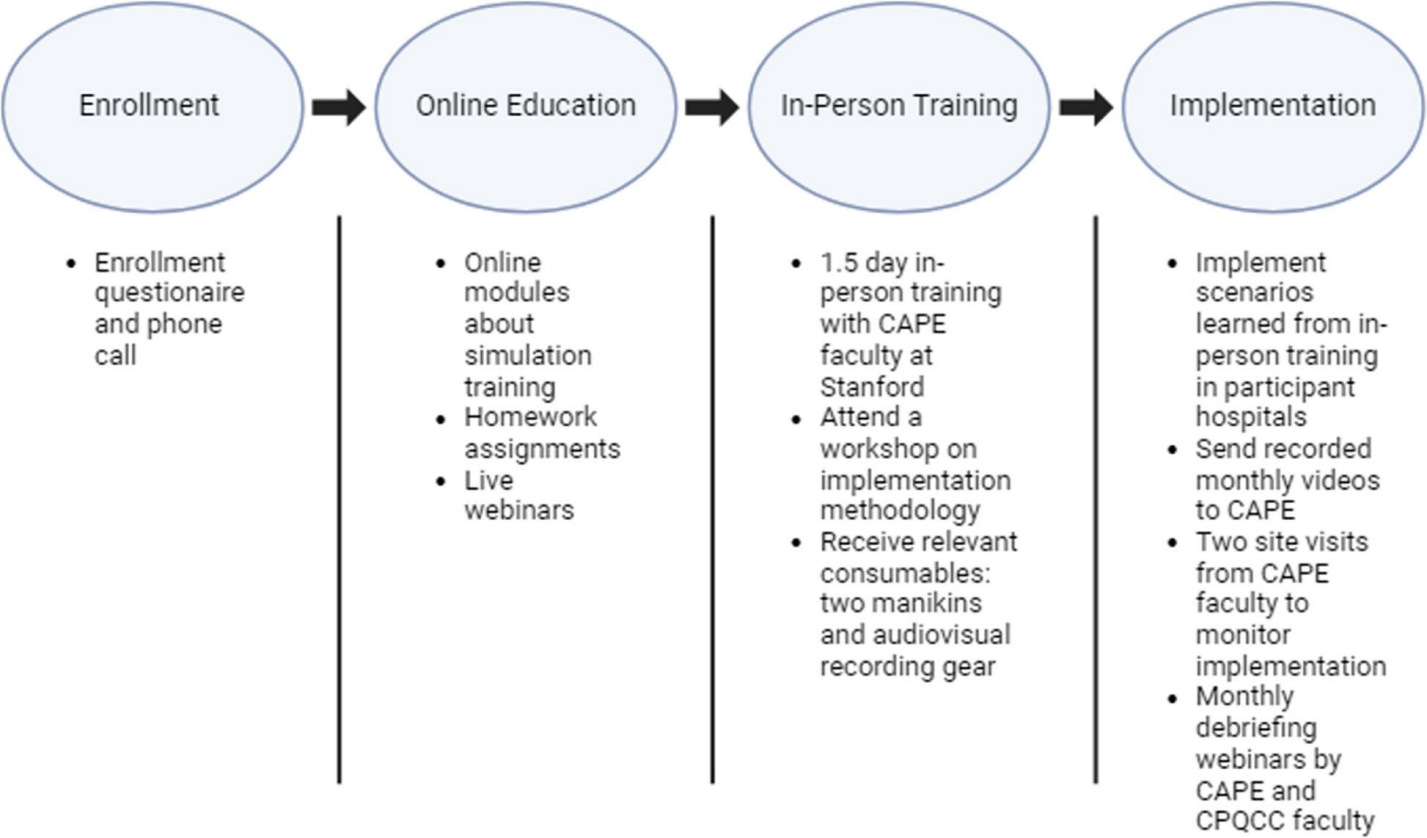
Simulating Success program structure. CAPE = Center for Advanced Pediatric & Perinatal Education; CPQCC = California Perinatal Quality Care Collaborative

The Simulating Success program was comprised of an education phase and an implementation phase. Figure 1 is a schematic illustration of the main program activities and timeline.

The education phase consisted of online webinars and learning modules on simulation-based training, followed by an in-person 1.5-day training program at the Center for Advanced Pediatric and Perinatal Education (CAPE) at Stanford. CAPE is a simulation-based training and research center that has been providing education for simulation instructors from all fields of healthcare for over 20 years. The online didactic curriculum was designed by CAPE and made available to an unlimited number of staff participants at each site shortly after the site’s registration for participation in Simulating Success. The in-person training was attended by a maximum of three staff members from each site. At the in-person training, each site was given two neonatal manikins (term and preterm) and basic audiovisual equipment with which to record simulations and debriefings during the implementation phase.

In the implementation phase (which was designed to take place over a 12-month period), site participants applied their learned techniques to design, conduct, and record neonatal resuscitation simulations and debriefings at their respective hospitals. Sites were instructed to upload all videos recorded that had been approved in regard to consent by participants to a shared, access-protected cloud storage drive on a monthly basis for quality review by CAPE faculty. CAPE faculty made two site visits to each participant hospital to observe their simulations and debriefings. In addition, monthly follow-up webinars were organized by CAPE and CPQCC faculty to review simulation strategies, debrief implementation thus far, and promote shared learning amongst participant hospitals.

### Participant sites

A total of 17 sites registered for the program, but three sites dropped out before initiating the first day of training. Of the 14 sites that remained involved after the first day of simulation training, four sites consistently completed data collection on resource utilization during their participation and were included in this analysis. All four sites were level 3 neonatal intensive care units (NICUs). More detail about the characteristics of these four included sites, in comparison to the other sites that were not included in the cost analysis, are reported in Appendix A.

### Cost data collection

We examined costs related to the design and implementation of the Simulating Success program for CPQCC and the four study sites using a micro-costing approach following a pre-specified economic analysis plan. Data collection forms were designed to prospectively track relevant resource utilization, including personnel time, equipment/supplies, space use, and travel (including transportation, food, and lodging). For each of these cost categories, we collected data on the quantity of resource use, as well as their corresponding unit cost (Table 1). Only resources used for the program itself were included, whereas resources used for research activities were excluded (e.g., personnel time spent on research data collection).

**Table 1:** Measurement and valuation of resource utilization

**Type of Resource**	**Quantity**		**Unit Cost**	
	**Measurement Unit**	**Data Source**	**Resource Valuation**	**Data Source**
Personnel time	Hour	Study records	Occupation-specific hourly wage rate	United States Bureau of Labor Statistics [11]; Doximity annual Physician Compensation Report [12–15]; Association of American Medical Colleges Survey of Resident and Fellow Stipends and Benefits [16]; The Physicians Foundation Survey of America’s Physicians [17]
Equipment and supplies	Item	Study records	Invoice/receipt price	Expense reports
Space use	Square footage; duration of use	Study records	Space-type specific annual per square foot leasing price	Authors’ discussion with study institution finance department
Travel (including transportation, food, and lodging)	Event	Study records	Invoice/receipt price	Expense reports; authors’ online search

To estimate personnel cost, we tracked the number and type of personnel involved, as well as the duration of time and date spent on various activities, such as designing training material, delivering online training, attending in-person sessions, and organizing local neonatal resuscitation training. For each type of personnel, we assigned a relevant occupation code based on the United States Bureau of Labor Statistics Standard Occupational Classification (SOC) codes whenever feasible and applied their corresponding national mean hourly wage rates [11]. Recognizing that neonatologists complete additional subspecialty training whereas the SOC occupation code does not distinguish neonatologists from general pediatricians, we used the hourly wage rate for neonatologists reported in the Doximity annual Physician Compensation Report [12–15]. Since there is no SOC occupation code for physician residents, we calculated hourly wage rate for residents based on compensation data from the Association of American Medical Colleges Survey of Resident and Fellow Stipends and Benefits and the mean number of work hours among physicians reported in the Survey of America’s Physicians conducted by The Physicians Foundation [16, 17]. Several student research assistants supported the delivery of the program (after excluding their hours spent on research); we used the standard undergraduate student wage scale at Stanford University.

For equipment and supplies, we tracked the use of various items over time, including the type and quantity of each item purchased, date of purchase, invoice or receipt price, and the proportion of its use for neonatal resuscitation training in general and for the Simulating Success program specifically (as opposed to other projects or for research). Consumables (such as power cords and cables purchased for use in simulation or debriefing) were assumed to be fully consumed during the program period; whereas for larger items, we assumed 10 years useful life for manikins and 5 years useful life for other devices (e.g., iPad, camera, and lens), and prorated their costs based on the duration of their use in the program and assuming linear depreciation.

For space use, we tracked each occasion of relevant room use and documented the purpose of use, the type and size of room used, date and duration of use, and the proportion of its use for neonatal resuscitation training in general and for the Simulating Success program specifically. We grouped space into two categories - clinical space (e.g., NICU patient room and labor and delivery patient room) versus general office space (e.g., staff office and conference room) - and applied the typical annual per square foot leasing price at our institution.

We also tracked expenses related to travel for the Simulating Success program, including air or ground transportation, food, and lodging for site participants to attend the in-person training sessions at CAPE and for the CPQCC team to deliver the in-person training sessions and site visits. We used the actual expense reports for these travel events whenever available and extracted information on the date and expenses of travel. When the source expense reports were not available, we used best estimates available through internet search.

**Fig. 2:**
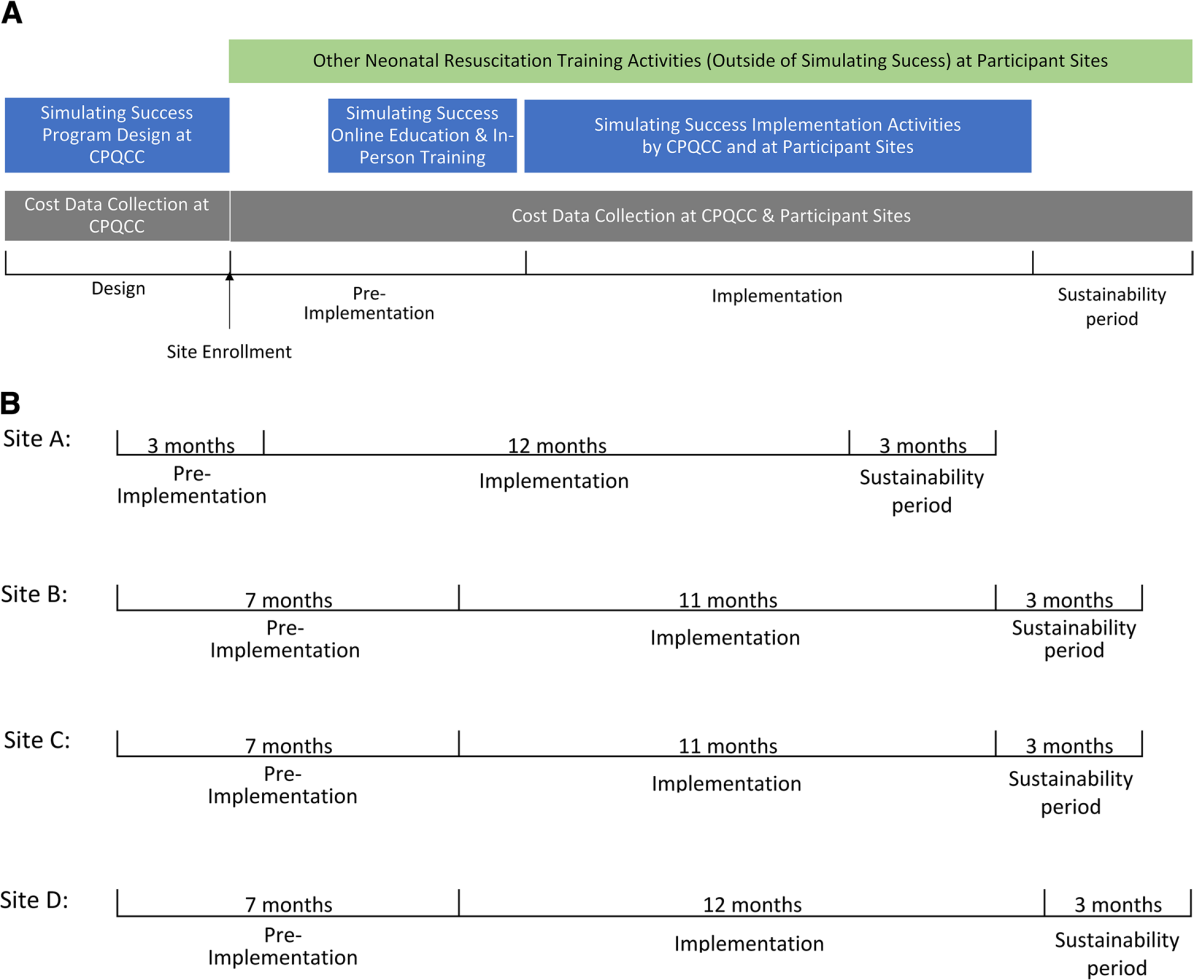
Illustration of cost data collection timeline. **A** Overall timeline. **B** Site-specific timeline

We collected the above data for all Simulating Success design activities for CPQCC, as well as all implementation activities by CPQCC and the four study sites during online education, in-person training, and implementation. In addition, we recognized that the Simulating Success program may substitute other neonatal resuscitation training activities at participant sites and therefore offset their overall cost. Moreover, resource utilization was expected to decrease after the intensive program activities ended (e.g., site visits) but sites could continue to benefit from the program. To inform these effects, we also collected data on resource use for other neonatal resuscitation training activities (outside of the Simulating Success program) from the date each site enrolled in the study until 3 months after the Simulating Success implementation ended (see data collection illustration in Fig. 2A). We referred to the months between site enrollment and start of implementation as pre-implementation period and the months after the end of the Simulating Success program as sustainability period.

### Cost analysis

To inform the impact on healthcare resource utilization, we estimated the cost of the Simulating Success program from a healthcare sector perspective. We first calculated the costs of designing and delivering the Simulating Success program incurred by CPQCC, as well as the costs of implementing the program incurred at each study site. These cost estimates were reported separately by site and by cost category. The time horizon included both program design and implementation.

We then calculated the program cost per site by equally allocating CPQCC cost across relevant sites and adding to site-specific program costs. For instance, CPQCC cost for the overall design of the program was allocated equally across 17 sites (the number of sites that initially registered for the program), while CPQCC cost for the delivery of group-based in-person training sessions were equally split across 14 sites (the number of sites that completed those in-person training sessions). We reported the program cost per site for each of the four study sites and summarized their mean and 95% confidence interval (CI). Since the period of program implementation for each site was close to one year, we did not perform discounting. All cost estimates were inflation adjusted to 2022 U.S. dollars using the medical care component of the Consumer Price Index [18].

Because of the high cost of manikins, in addition to assuming a 10-year useful life in our primary analysis, we ran a sensitivity analysis by varying the useful life of a manikin from 5 to 12 years. To inform the cost implications of scaling up the program to more sites, we also conducted sensitivity analyses for the following three scenarios: 1) assuming 30 sites participated and shared the program design costs, 2) assuming 50 sites participated and shared the program design costs, and 3) excluding program design cost to emulate situations where a mature program was directly adopted and implemented.

In addition, we estimated the cost of other neonatal resuscitation training (outside of the Simulating Success program) at each site during the implementation period and compared it with baseline neonatal resuscitation training cost in the pre-implementation period to inform the potential substitution effect of the Simulating Success program. Furthermore, we estimated the cost of all neonatal resuscitation training activities at each site in the sustainability period and compared it with such cost in the pre-implementation period to inform whether Simulating Success program might produce financial benefit in the long run (i.e., after the acute intervention period). To facilitate the comparison of cost estimates across study sites (because the exact period of Simulating Success implementation was 11 months at some sites but 12 months at others for logistical reasons during program operation) and across study periods (because a site might have cost data for a 7-month pre-implementation period, 12-month implementation period, and 3-month sustainability period) (see Fig. 2B), we reported these cost estimates in mean monthly values.

Data analysis was completed using SAS version 9.4 (SAS Institute, Cary, NC). We followed the Consolidated Health Economic Evaluation Reporting Standards 2022 [19].

## Results

Table 2 summarizes the breakdown of Simulating Success program cost. The cost for designing and delivering the program totaled $228,148.36 for CPQCC. Personnel cost accounted for the largest share of the CPQCC cost (92.2%), followed by program-related travel (including transportation, food, and lodging) (6.1%), equipment/supplies (1.5%), and space use (0.2%). Cost for implementing the program at the four study sites varied from $18,056.60 to $30,326.27. Distribution of the cost categories was relatively similar, with personnel cost accounting for 77.8%-87.1% of the site-specific costs. Site A had the highest implementation cost and used a total of 534.5 person-hours, whereas site B had the lowest implementation cost and used a total of 233.0 person-hours (Table 3).

**Table 2:** Breakdown of Simulating Success program cost by cost category and study site

**Cost Category**	**CPQCC**^**a**^	**Site A**	**Site B**	**Site C**	**Site D**
Personnel time					
Program design	$57,016.64	NA	NA	NA	NA
Online and in-person training	$49,672.43	$7,260.46	$4,811.59	$5,637.38	$6,694.50
Program support/implementation	$103,661.16	$17,372.35	$9,245.16	$15,626.15	$12,035.13
Equipment and supplies					
Standard set of program equipment	NA	$800.89	$800.89	$800.89	$800.89
Other equipment and supplies	$3,443.34	$542.16	$197.94	$0	$68.43
Space use	$454.81	$256.47	$42.44	$331.40	$163.41
Travel (transportation, food, and lodging)	$13,899.98	$4,093.94	$2,958.58	$2,413.93	$1,750.92
Total cost	$228,148.36	$30,326.27	$18,056.60	$24,809.75	$21,513.28

Numbers may not add up exactly due to rounding

*CPQCC* California Perinatal Quality Care Collaborative, *NA* Not applicable

^a^Include costs incurred by the CPQCC in managing the program for all relevant sites

**Table 3:** Personnel type and time involved in the design and implementation of the Simulating Success program by study site

**Site**	**Type of Personnel**	**Person-Hours Involved**
CPQCC	Neonatologist	1047.0
	Nurse practitioner	422.7
	Registered nurse	30.0
	Other healthcare practitioner and technical occupation	35.5
	Statistician	2.6
	Technical writer	3.0
	Research assistant	1650.7
	Student research assistant	264.3
	***Subtotal***	***3455.8***
Site A	Neonatologist	41.0
	Registered nurse	377.5
	Respiratory therapist	116.0
	***Subtotal***	***534.5***
Site B	Neonatologist	53.5
	Registered nurse	145.5
	Respiratory therapist	29.0
	Other healthcare practitioner and technical occupation	5.0
	***Subtotal***	***233.0***
Site C	Neonatologist	42.5
	General pediatrician	5.0
	Registered nurse	272.0
	Respiratory therapist	105.0
	Resident	2.0
	***Subtotal***	***426.5***
Site D	Neonatologist	51.0
	Other physician	6.0
	Registered nurse	222.5
	Respiratory therapist	55.5
	***Subtotal***	***335.0***

Numbers may not add up exactly due to rounding

*CPQCC* California Perinatal Quality Care Collaborative

After allocating all CPQCC-incurred program design and delivery costs to relevant participant sites, the cost of the Simulating Success program averaged $39,210.69 per site (95% CI: $34,094.52–$44,326.86) (Table 4). In sensitivity analyses varying the useful life of manikins from 5 to 12 years, the estimated mean cost became $39,935.73 per site and $39,089.84 per site, respectively. If the program was expanded to 30 or 50 sites, the estimated mean cost would be $37,669.55 per site and $36,863.42 per site, respectively. If the program was taken as is and directly adopted at other sites (i.e., disregard the program design cost), the estimated mean cost would be $35,645.22 per site.

**Table 4:** Cost of Simulating Success program per site

**Analysis**	**Mean Cost per Site (95% Confidence Interval)**
Primary analysis	$39,210.69 ($34,094.52–$44,326.86)
Sensitivity analysis	
Assume 5-year useful life for manikin	$39,935.73 ($34,592.14–$45,279.33)
Assume 12-year useful life for manikin	$39,089.84 ($34,011.22–$44,168.47)
Assume 30 participant sites	$37,669.55 ($32,553.38–$42,785.72)
Assume 50 participant sites	$36,863.42 ($31,747.25–$41,979.59)
Assume no program design cost	$35,645.22 ($30,538.05–$40,770.39)

All costs are reported in 2022 U.S. dollars

The Simulating Success program appeared to substitute some of the neonatal resuscitation training needs at participant sites. This was evidenced by a lower monthly cost of other neonatal resuscitation training during the program implementation period (mean: $1,112.52 per site) than the baseline pre-implementation period (mean: $2,504.01 per site) at all four sites (Table 5).

**Table 5:** Average monthly cost of neonatal resuscitation training by study site and implementation period

**Site**	**Number of NICU Beds**^**a**^	**Pre-Implementation Period**	**Implementation Period**		**Sustainability Period**
		**All Neonatal Resuscitation Training**	**Simulating Success Program**^**b**^	**Other Neonatal Resuscitation Training**	**All Neonatal Resuscitation Training**
Site A	> 51	$2,346.11	$3,821.71	$1,569.80	$4,191.34
Site B	< 22	$317.65	$3,053.71	$128.74	$384.36
Site C	22–51	$1,057.85	$3,667.63	$780.30	$693.80
Site D	22–51	$6,294.42	$3,087.29	$1,971.22	$2,590.96
Mean (95% CI)	NA	$2,504.01 ($244.53–$4,763.48)	$3,407.59 ($3,072.76–$3,742.41)	$1,112.52 ($415.30–$1,809.73)	$1,965.12 ($457.82–$3,472.41)

All costs are reported in 2022 U.S. dollars

*CI* Confidence interval, *NA* Not applicable, *NICU* Neonatal intensive care unit

^a^To protect confidentiality of study sites, number of NICU beds was reported in categories

^b^Included the costs of online education and in-person training, as well as California Perinatal Quality

Care Collaborative (CPQCC) costs of program design and implementation that were allocated to relevant participant sites

In the sustainability period immediately after the Simulating Success program ended, monthly cost of all neonatal resuscitation training averaged $1,965.12 per site, which was also lower than the average monthly cost in the pre-implementation period (Table 5). However, there was large variability across the four sites resulting in a large 95% CI ($457.82–$3,472.41). The monthly cost of neonatal resuscitation training was lower in the sustainability period than in the pre-implementation period at two sites but higher at the other two sites.

## Discussion

Reports on the implementation of simulation programs in various settings are ample in the realm of healthcare, with many suggesting cost-effective results [20–23]. However, studies reporting simulation implementation specifically within the NICU and its associated cost are sparse. Our study addressed this gap by presenting data on the Simulating Success program, which was designed to promote and facilitate simulations at multiple hospitals with the goal of improving local patient outcomes by addressing teamwork and local systems issues for practicing NICU healthcare professionals. Our findings inform the financial and logistical requirements for completing a collaborative of this magnitude and scope.

It should be noted that establishing a multi-site program across multiple sites in a state as large as California is a difficult task to accomplish. Expectations for the central design team included significant time investment from personnel, meetings to communicate needs and progress with participant sites, development of a training curriculum (online and in-person), provision of supplies and equipment, and appropriate coordination of space use and travel for in-person training and site visits. For instance, the design, training, and subsequent program support required a total of 3,455.8 person-hours from a diverse team of CPQCC staff and collaborators (e.g., neonatologists, nurse practitioners, registered nurses, research assistants, and a technical writer). Execution of the Simulating Success program benefited from the bandwidth of CPQCC and its well-established network within California. Nevertheless, the needs and expectations of participant sites are often diverse, and the most effective simulation-based training programs need to be tailored to the needs of each specific sites. Within the context of Simulating Success, the central design team drew on CAPE’s pre-existing curriculum and adapted it via close collaboration with CAPE faculty. Therefore, when developing future multi-site simulation implementation programs of this scale, adequate capacity of the central design team that implements initial facilitation should be considered to ensure project completion.

The varying cost of implementing the Simulating Success program across participant sites likely reflect variation in the complexity of personnel involved (e.g., number and type of healthcare professionals) and the needs for neonatal resuscitation training at each site. In particular, the number of NICU beds varied by six-fold across the four study sites included in our analysis which may signal different personnel needs. The cost of implementing the Simulating Success program was generally higher at sites with more NICU beds, whereas participant sites that had fewer NICU beds tended to incur lower costs even in the pre-implementation period. For instance, the Simulating Success program costed $3,053.71 per month at the site with the fewest NICU beds to $3,821.71 per month at the site with the most NICU beds. Sites with different NICU sizes and staffing structures likely differ in the type and number of providers involved in neonatal resuscitation training activities and the intensity of their training activities. Further research comparing different structures of the training team and modalities (e.g., amount of virtual versus in-person training, frequency and size of simulation sessions, and composition of core team) will help inform ways to reduce costs while preserving the clinical benefit of the program.

Although the design and delivery of the Simulating Success program itself involved high resource utilization, it may have financial benefits in the long run. For instance, the program activities helped offset the need for some of the other neonatal resuscitation training as shown in our data. Two of the study sites had a lower monthly cost of overall neonatal resuscitation training in the sustainability period than in the pre-implementation period. Although we do not know the exact type of training activities that was occurring at participant sites prior to the Simulating Success program, it presumably involved some standard neonatal resuscitation training which does include simulation albeit not in situ simulation. This is consistent with other studies demonstrating the cost-neutral effect of an in situ simulation model using minimal permanent space and redirected faculty educational time [20]. While the unpredictable nature of NICU workflow and patient acuity can sometimes make in situ simulations challenging, integrating practice into regular work shifts may enhance efficiency and also more accurately reflect how teams would work in real life. Having the experienced CAPE team that had already refined strategies of simulation and debriefing for neonatal resuscitation may have reduced the overall cost. If there can be sustained benefit in improved efficiency in training and practice, the high program cost may be further offset in the long run. Moreover, our sensitivity analysis suggests that the high cost of Simulating Success program design can be more economical if there are more participant sites to share it.

In addition to the financial impact, it is also important to consider the program’s effect on improving clinical practice. Research on the Simulating Success program showed that although it did not improve neonatal survival without chronic lung disease, the program may have an impact on unit practice [10]. In addition to improving clinicians’ technical and behavioral skills, participant sites were able to holistically benefit from the program, with many participants reporting improved team participation, ability to identify latent safety threats, and process/system changes within respective NICUs [1, 9, 10, 24]. In focus group discussions, neonatal healthcare professionals at participant sites reported that the Simulating Success program helped provide an environment in which clinicians felt less punitive and safe to describe team performance and team communication, which can facilitate culture change in the unit [24]. Future studies evaluating the cost-effectiveness of such programs should consider these benefits in clinical practice and system improvements in addition to patient outcomes.

We recognize several limitations of this study. First, out of the 14 sites that completed initial training, we only had adequate cost data from four of them. This exemplifies the challenges in collecting cost data for implementation projects in busy clinical settings. For instance, accurate tracking of cost information may involve data collection approaches that participants are less accustomed to, activities that are above their typical workload, and information that may not be readily documented/available (e.g., extracting nuanced information from invoices, documenting specifics about meeting durations and room sizes, and recording details about attendee credentials and transportation). When there is limited staff, clinical care and actual implementation of the simulation program would take priority over these data collection activities. Therefore, experience on the financial impact of the four included sites may not be generalizable to all participant sites, especially given the larger NICU size at these four sites in comparison to other participant sites (median: 48 NICU beds versus 25 NICU beds). As the volume of training activities and staff involved may be smaller in scale at NICUs with fewer beds, it is possible that if costs from all participant sites were analyzed, the average program cost per site would be lower than our estimates. Likewise, our findings may not be generalizable elsewhere in the country because the Simulating Success program was implemented in California. However, whenever feasible, we used national mean unit prices in cost estimation (e.g., mean hourly wage rates for personnel time which accounted for the largest share of the program cost). Second, some participant sites had limited healthcare team availability and high turnover in key roles which was time-consuming in conducting simulations and re-training [10]. As a result, our data likely overestimated the cost and underestimated the efficacy of the Simulating Success program. Third, although we included a sustainability period and collected cost data in the three months immediately after the program ended, the longer-term impact of the Simulating Success program remains unknown. It is uncertain whether the intensive training and support provided during the program had a lasting effect on provider practice and teamwork over time. Longer-term follow-up data would be helpful to further inform the financial and clinical benefits of the program. Finally, all NICUs participating in the Simulating Success program were located in urban areas. Although we anticipate the impact of the program on NICUs in rural areas would be similar to sites with fewer NICU beds in our sample, future research formally evaluating such programs in rural NICUs will be instrumental.

## Conclusions

Establishing a multi-site neonatal in situ simulation program requires an investment of sufficient resources, personnel, and time from design and participant sites. This should be adequately recognized and carefully considered prior to project initiation. However, such programs have the potential to improve simulation-based training and bring positive changes in practice.

### Supplementary Information

Supplementary Material 1.

## Data Availability

The datasets generated and/or analyzed during the current study are not publicly available due to privacy restrictions.

## Abbreviations

CAPE: Center for Advanced Pediatric and Perinatal Education
CI: Confidence interval
CPQCC: California Perinatal Quality Care Collaborative
NA: Not applicable
NICU: Neonatal intensive care unit
SOC: Standard Occupational Classification
