# Supplementary material for: Implementation of a multi-site neonatal simulation improvement program: a cost analysis
Source: BMC Health Serv Res. 2024 May 14;24:623. doi: 10.1186/s12913-024-11075-z (PMC11090826; doi:10.1186/s12913-024-11075-z)
Supplement: Supplementary file 1 — Supplementary Material 1. [file 12913_2024_11075_MOESM1_ESM.docx]

**Appendix**

**Appendix A. Comparison of sites included versus excluded from the analysis**

| **Characteristics** | **Overall**  **(N=17)** | **Completed program and included in analysis**  **(n=4)** | **Participated in program but later withdrew or lacked adequate cost data**  **(N=10)** | **Dropped out before initiating first day of training**  **(N=3)** |
| --- | --- | --- | --- | --- |
| NICU level |  |  |  |  |
| Level 3 | 15 (88.2%) | 4 (100%) | 8 (80%) | 3 (100%) |
| Level 4 | 2 (11.8%) | 0 (0%) | 2 (20%) | 0 (0%) |
| Number of NICU beds |  |  |  |  |
| Median (IQR) | 27  (22-51) | 48  (37.25-59.25) | 25  (22.5-46) | 27  (23.5-29.5) |
| Teaching status |  |  |  |  |
| Teaching | 4 (23.5%) | 1 (25%) | 3 (30%) | 0 (0%) |
| Non-teaching | 13 (76.5%) | 3 (75%) | 7 (70%) | 3 (100%) |
| Urban-Rural location |  |  |  |  |
| Urban | 17 (100%) | 4 (100%) | 10 (100%) | 3 (100%) |
| Rural | 0 (0%) | 0 (0%) | 0 (0%) | 0 (0%) |
| Type of ownership |  |  |  |  |
| Government, non-federal | 2 (11.8%) | 1 (25%) | 1 (10%) | 0 (0%) |
| Private for-profit | 2 (11.8%) | 1 (25%) | 1 (10%) | 0 (0%) |
| Private non-profit | 13 (76.5%) | 2 (50%) | 8 (80%) | 3 (100%) |

IQR = interquartile range; NICU = neonatal intensive care unit.

Out of the 17 sites registered for the Simulating Success program, 3 sites dropped out before initiating the first day of training; 10 sites remained involved after the first day of training but were excluded from this analysis because they later withdrew from the program or lacked adequate cost data; and 4 sites completed the program, consistently collected cost data, and were included in this analysis.
